# Interprofessional Healthcare Education on Aging Increases Students' Confidence in Medication Management Skills

**DOI:** 10.1093/geroni/igaa057.045

**Published:** 2020-12-16

**Authors:** Laura Finn, Deborah Summers

**Affiliations:** University of the Sciences, Philadelphia, Pennsylvania, United States

## Abstract

Students studying for health care professions have limited opportunities to learn about medication use and aging in an interprofessional experience. Health Care students who interact in a simulation of age-related sensory changes can identify adaptations for safe medication use and counseling necessary to promote healthy aging. This research assessed the impact of a simulated team experience on pharmacy and physician assistant students’ confidence in understanding age related changes and in learning adaptations to promote safe medication use for older adults who may experience those changes. 63 pharmacy and 113 Physician Assistant students participated in 2-hour Interprofessional Education (IPE) sessions. The teams of pharmacy/physician assistant students utilized glasses to simulate changes in vision and gloves to simulate conditions of arthritis and neuropathy which increase in prevalence with age. Teams practiced skills of medication counseling and empathy towards their peers experiencing the simulations and learned medication administration adaptations for aging well. Pre survey results show a deficit of Pharmacist-Physician Assistant IPE with less than 20% of students reporting a strong understanding of the other profession’s role in developing an older adult’s care plan. Post survey results demonstrate an increase in students’ confidence in both understanding how sensory impairments may affect a patient’s ability to properly administer medication and confidence in counseling older adults on safe medication use. Descriptive data on learning in Interprofessional teams, Pre/Post comparison data and application to students studying other majors will be presented.

